# Microsatellite DNA Analysis Revealed a Drastic Genetic Change of *Plasmodium vivax* Population in the Republic of Korea During 2002 and 2003

**DOI:** 10.1371/journal.pntd.0002522

**Published:** 2013-10-31

**Authors:** Moritoshi Iwagami, Seung-Young Hwang, So-Hee Kim, So-Jung Park, Ga-Young Lee, Emilie Louise Akiko Matsumoto-Takahashi, Weon-Gyu Kho, Shigeyuki Kano

**Affiliations:** 1 Department of Tropical Medicine and Malaria, Research Institute, National Center for Global Health and Medicine, Shinjuku-ku, Tokyo, Japan; 2 Department of Parasitology, Inje University College of Medicine, Busanjin-gu, Busan, Korea; 3 Department of Infectious Disease and Malaria, Paik Institute of Clinical Research, Inje University, Busanjin-gu, Busan, Korea; 4 Department of Community and Global Health, Graduate School of Medicine, The University of Tokyo, Bunkyo-ku, Tokyo, Japan; National Institute of Parasitic Diseases, China

## Abstract

**Background:**

Vivax malaria was successfully eliminated in the Republic of Korea (South Korea) in the late 1970s, but it was found to have re-emerged from 1993. In order to control malaria and evaluate the effectiveness of malaria controls, it is important to develop a spatiotemporal understanding of the genetic structure of the parasite population. Here, we estimated the population structure and temporal dynamics of the transmission of *Plasmodium vivax* in South Korea by analyzing microsatellite DNA markers of the parasite.

**Methodology/Principal Findings:**

We analyzed 14 microsatellite DNA loci of the *P. vivax* genome from 163 South Korean isolates collected from 1994 to 2008. Allelic data were used to analyze linkage disequilibrium (LD), genetic differentiation and population structure, in order to make a detailed estimate of temporal change in the parasite population. The LD analysis showed a gradual decrease in LD levels, while the levels of genetic differentiation between successive years and analysis of the population structure based on the Bayesian approach suggested that a drastic genetic change occurred in the South Korean population during 2002 and 2003.

**Conclusions/Significance:**

Although relapse and asymptomatic parasite carriage might influence the population structure to some extent, our results suggested the continual introduction of *P. vivax* into South Korea through other parasite population sources. One possible source, particularly during 2002 and 2003, is North Korea. Molecular epidemiology using microsatellite DNA of the *P. vivax* population is effective for assessing the population structure and temporal dynamics of parasite transmission; information that can assist in the elimination of vivax malaria in endemic areas.

## Author Summary

Vivax malaria is widely prevalent, predominantly in Asia and South America. There were 390 million cases reported in 2009. Worldwide, in the same year, 2.85 billion people were at risk of infection. *Plasmodium vivax* is not limited to tropical and subtropical regions; it also appears in temperate areas. We previously examined the characteristics of *P. vivax* in South Korea, a temperate area, temporally, using 10 microsatellite DNA (a short tandem repeat DNA sequence) in the parasite genome and found the population to have low genetic diversity and a low recombination rate in comparison to tropical areas. In the present study, we examine the successive changes of the South Korean populations from 1994 to 2008, and the reasons for the lack of success in eliminating vivax malaria in this country. We found that, in spite of a low recombination rate, outbreeding between different genotypes seemed to increase gradually, and that the genetic composition of the population drastically changed during 2002 and 2003. This suggests that the parasite is continually introduced from other populations, probably from North Korea. The present study also demonstrated the utility of polymorphic DNA markers of *P. vivax* for the estimation of the transmission situation in endemic areas.

## Introduction

*Plasmodium vivax*, which is the second most prevalent species of the human malaria parasite, is widely distributed around the world especially in Asia, Melanesia, the Middle East, South and Central America. There are 390 million cases reported annually and, as of 2009, there were 2.85 billion people at risk of infection [1], [2]. Treatment and/or prophylactic failures of chloroquine to vivax malaria have been reported from several endemic countries since the late 1980s [3]. Moreover, treatment failures of primaquine to hypnozoites of *P. vivax* have also been reported, although it should be noted that it is difficult to discriminate between reinfection and parasite tolerance [3]. Severe cases of vivax malaria were also reported in endemic areas [4], [5]. Thus, *P. vivax* deserves more attention than it has previously received [6].

Understanding the genetic characteristics of the malaria parasite population is important for the monitoring of the transmission pattern and evaluation of the effectiveness of malaria control in endemic areas [7]–[10]. The transmission dynamics and population structures of *P. vivax* have recently been reported in some tropical and subtropical areas where the parasites are prevalent year-round or prevalent in a particular season but continuous during the year [11]–[19]; much less is known, however, about these characteristics in temperate areas where vivax malaria is seasonally prevalent but not continuous throughout the year [20]–[25].

In the Republic of Korea (South Korea), which is located in the temperate zone of Asia, indigenous vivax malaria was successfully eliminated by the late 1970s due to an effective program implemented by the National Malaria Eradication Service of the South Korean government with support from the WHO [26]–[28]. There has, however, been a re-emergence since 1993 [29]. The only patients at the beginning of the re-emergence were soldiers from the South Korea and the United States or veterans who had served in the western Demilitarized Zone, which is in the border area between the Democratic People's Republic of Korea (North Korea) and South Korea [30]–[32]. However, the number of infected civilians who lived in or near the area gradually increased [30], which suggested that *P. vivax* was being transmitted locally between humans and *Anopheles* mosquitoes. In spite of continuous malaria control measures implemented by the South Korean government, there was a steady increase in the number of reported vivax malaria cases until 2000 (4,183 cases), then a gradual decrease until 2004 (864 cases) [1], [28]. The number of reported cases fluctuated between 838 and 2,227 per year from 2005 to 2011 [1]. Vivax malaria was considerably more prevalent in North Korea, with 300,000 cases in 2001 and 14,845 cases in 2009 [1], [31].

In a previous study, we assessed the population structure and temporal dynamics of *P. vivax* transmission in South Korea using the allelic data pertaining to 10 microsatellite DNA loci of 87 isolates (1994–2008), and highlighted the differences between the parasite populations in tropical and temperate regions [33]. We found a gradual increase in the levels of genetic diversity and a decrease in the levels of multilocus linkage disequilibrium (LD) from 1994 to 2008. Choi et al. [34] and Honma et al. [35] reported similar tendencies. Furthermore, we found that two main groups of haplotypes had been transmitting for nearly 10 years. This suggested that in temperate South Korea, the recombination rate of *P. vivax* was lower than in tropical areas in which genetically identical haplotypes (clones) were seldom seen in two consecutive years. Despite the low recombination rate, other new haplotypes have been observed since 1997. These new haplotypes are genetically distinct from the main two. Taken together, these results suggest that *P. vivax* is being continually introduced from other population sources.

Our previous study was limited by its relatively small sample size per year (average: 5.8 isolates/year) and some of the sample sizes during the year 2004–2008 were particularly small (2 to 7 isolates, average: 3.6 isolates/year). This was, perhaps, too small to discuss the temporal dynamics of parasite transmission in detail. Thus, in the present study, we increased the number of microsatellite DNA loci (from 10 to 14 loci) as well as the sample size of *P. vivax* parasites (average: 10.9 isolates/year) from South Korea, and performed population genetic analysis, with a focus on the differences of the parasite populations between successive years. Through this, we aimed to provide a more detailed and precise estimate of the characteristics of the vivax malaria population structure and the temporal dynamics of its transmission. Based on our new findings, we discuss the situation of the *P. vivax* population in South Korea and provide a possible explanation as to why, in spite of a continuous malaria control program, efforts to eliminate vivax malaria have been unsuccessful.

## Methods

### Ethics statement

Ethical approval for the use of South Korean *P. vivax* samples in this study was obtained from the Inje University Busan Paik Hospital Institutional Review Board, Busan, Korea, and performed in accordance with the Ethical Guidelines for Clinical Research issued by the Ministry of Health, Labour and Welfare of Japan on July 31, 2008, and the Ethical Guidelines for Epidemiological Research issued by the Ministries of Health, Labour and Welfare, and of Education, Science, Culture, and Sports of Japan on December 1, 2008. Written or oral informed consent from the patients could not be obtained at each sample collection for the specific purpose of this study because of the long-term prior collection of widely distributed samples. However, no author of the study was involved in gathering patient samples and the donors' personal information was disconnected from the authors. Thus, all of the samples were anonymous and it is doubtful that the analysis of the results would constitute a breach of donor privacy.

### Materials

Two hundred and fifty-five *P. vivax* samples isolated from South Korean soldiers or veterans who had served in the DMZ from 1994 to 2008 were used in the present study. These cases were also diagnosed through microscopic examination of peripheral blood smears after the patients contracted malaria. Blood samples were collected and preserved until use at −30°C.

### DNA extraction

Parasite DNA was extracted from frozen whole blood samples by phenol-chloroform extraction after proteinase K digestion [36] or by QIAamp DNA Mini Kit (Qiagen, Valencia, CA, USA) in accordance with the manufacturer's instructions.

### Genotyping by polymerase chain reaction (PCR)

Fourteen microsatellite DNA loci were amplified by PCR. The loci were as follows: MS1 (chromosome 3), MS2 (chromosome 6), MS3 (chromosome 4), MS4 (chromosome 6), MS5 (chromosome 6), MS6 (chromosome 11), MS7 (chromosome 12), MS8 (chromosome 12), MS9 (chromosome 8), MS10 (chromosome 13), MS12 (chromosome 5), MS15 (chromosome 5), MS16 (chromosome 9) and MS20 (chromosome 10) [37]. The ID of each *P. vivax* chromosome (Salvador-1 strain) is shown in Table S1. Four of the 14 microsatellite loci were new markers (MS2, MS3, MS10 and MS16), and were not used in the previous study [33]. PCR primer sets and amplification conditions were consistent with the protocol established by Karunaweera et al. [37]. Fluorescence-labeled PCR products were measured using an Applied Biosystems Prism Genetic Analyzer 3130*xl* with GeneMapper^(R)^ version 4.1 and a 500 ROX size standard (Applied Biosystems, CA, USA).

Amplified different-sized PCR products that used the same primer sets were considered to be individual alleles within a locus, because the variation in size among isolates was consistent with the repeat number in a microsatellite locus [8]. The electropherogram shows peak profiles for the microsatellite loci, based on the fluorescence intensity of the PCR products labeled in this analysis. Multiple alleles per locus were scored if minor peaks were taller than at least one-third of the height of the predominant allele for each locus on electropherograms. Multiple-genotype infections (MGIs) were defined as those in which at least one of the 14 loci contained more than one allele [8].

### Multilocus linkage disequilibrium of the *P. vivax* population in South Korea

Multilocus linkage disequilibrium (LD) was assessed based on the allelic data of 14 microsatellite DNA loci, using the standardized index of association (*I*_A_^S^) [38], [39]. This analysis was performed using the LIAN 3.5 Web interface software [40]. *I*_A_^S^ was calculated with the formula *I*_A_^S^ = (*V*_D_/*V*_e_−1)/(*l*−1), with permutation testing of the null hypothesis of the complete linkage equilibrium (*I*_A_^S^ = 0), where *V*_D_ is the observed mismatch variance, *V*_e_ is the expected mismatch variance and *l* is the number of examined loci. Significance levels of the observed *I*_A_^S^ values were calculated by Monte-Carlo simulation, using 10,000 random data permutations. This statistical method is a variation of that proposed by Smith et al. [38]. The results were standardized by the number of loci in order to enable a comparison of different data sets [33]. This test was applied to the whole data sets in two ways: 1) all haplotype data of the 163 isolates were analyzed, thereby providing complete confidence in the haplotype profile; 2) multilocus genotypes found in multiple isolates were only counted once in the analysis, such that only unique haplotypes were counted, slightly reducing the sample size and removing the possible effect of recent epidemic expansion of particular clones [8].

### Genetic differentiation of the *P. vivax* populations in South Korea between successive years

The extent of population subdivision for all pairs of years in South Korea was estimated based on the allelic data of 14 microsatellite DNA loci, using Weir and Cockerham's Ø estimator for determining *F* statistics (*F*_ST_) [41]. *F*_ST_ was calculated using the software FSTAT version 2.9.3.2 and tested for significant difference from 0, based on 1,000 random permutations of the data set [42].

### Population structure analysis of the South Korean *P. vivax* populations by Bayesian approach

The *P. vivax* population structure in South Korea (1994–2008) was estimated based on the allelic data of 14 microsatellite DNA loci using the Bayesian approach model with STRUCTURE version 2.3.4 software [43]. This clustering method assigns samples to the true number of clusters (*K*) according to allele frequencies of each locus. Structure analysis was performed with 10 runs for each of 10 different *K* values (1 to 10), with a length of 50,000 burn-in periods followed by 100,000 Markov Chain Monte Carlo replications. The admixture model, with the assumption of both correlated and uncorrelated allele frequencies among the populations, was used in this analysis. Population numbers were inferred by plotting the log probability of the data [Ln P(D)] for each *K* value. Moreover, in order to estimate the most appropriate number of *K* (true *K*), Δ*K*, which is the rate of change in the log probability of the data between successive *K* values, was calculated as described by Evanno et al. [44].

## Results

### Genotyping of allelic data

The allelic data of the 14 microsatellite loci were obtained from 163 of the 255 (63.9%) isolates from 1994 to 2008 (average: 10.9 isolates/year) that were used in the study. Allelic data of the 14 loci from the remaining 92 isolates (36.1%) were unavailable or only partially available due to failure in acquiring PCR products from some loci by PCR-based genotyping. Failure was possibly due to there only being a small amount of DNA for PCR amplification or because the quality of the DNA was low after multiple incidences of freeze-thaw.

**Figure 1:**
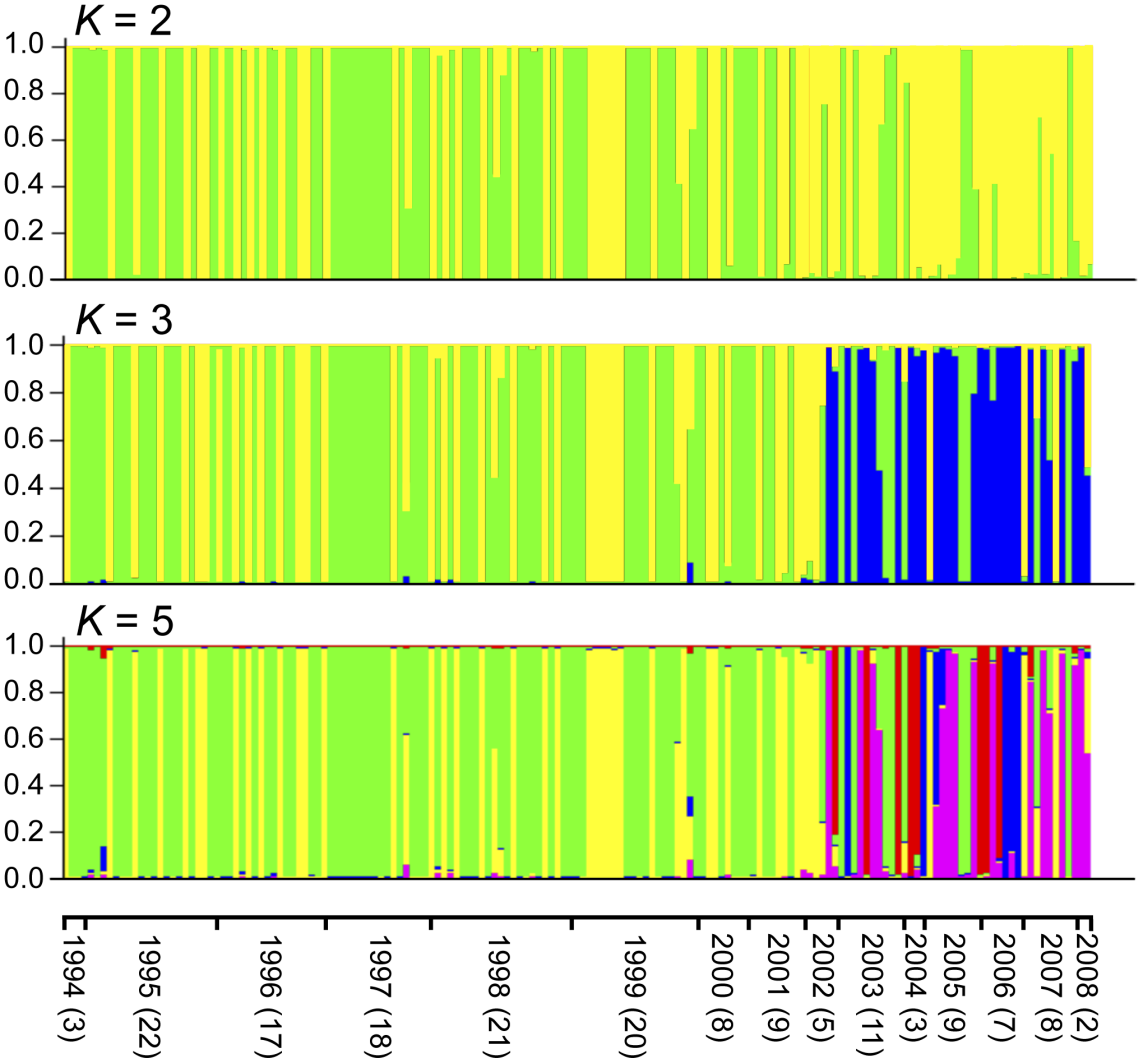
Estimated population structure of the South Korean *P. vivax* populations from 1994 to 2008. The population structure of the South Korean *P. vivax* population (163 isolates) from 1994 to 2008 was estimated by Bayesian approach using STRUCTURE software version 2.3.4, under the assumption of uncorrelated allele frequencies model [43]. Each isolate was represented by a vertical line partitioned into colored segments in proportion to the estimated membership (ancestral population). Different colors represent different genotypes (ancestral population). If one bar has more than two colors, it indicates that the genotype of the isolate is admixed with more than two ancestral populations. Results shown were for *K* = 2, 3 and 5. The numbers in parentheses after collection year represent the number of isolates for each year.

Figure 1 shows the number of isolates found in samples from each year (Fig. 1). In total, 71 unique haplotypes were observed in the 163 isolates (Table 1). The allelic composition of each haplotype is shown in Table S2. There were 2 major haplotypes: H35 and H43. H35 was observed in 22 (13.5%) of the 163 isolates in samples collected from 1995 to 2001. H43 was observed in 58 (35.6%) of the 163 isolates in samples collected from 1994 to 2001, 2003 and 2007. By increasing the number of loci (from 10 to 14) and isolates (from 87 to 163), the number of unique haplotypes was increased from 40 to 71 [33], which enabled us to perform a more precise estimation of the transmission dynamics of the South Korean *P. vivax* population over the study period. When compared to the previous study, however, the changes in the levels of genetic diversity (heterozygosity) observed in the present study were relatively minor (Table S1, Fig. S1 and S2) [33].

**Table 1:** Seventy-one haplotypes of the South Korean *P. vivax* population based on 14 microsatellite DNA loci.

H	Year															Total
	1994	1995	1996	1997	1998	1999	2000	2001	2002	2003	2004	2005	2006	2007	2008	
**H1**		1														1
**H2**			1													1
**H3**		1														1
**H4**		1														1
**H5**		1														1
**H6**							1									1
**H7**		1	1			1	1	1								5
**H8**												1				1
**H9**						1										1
**H10**	1															1
**H11**													1			1
**H12**										1						1
**H13**												1				1
**H14**													1			1
**H15**													1			1
**H16**											1					1
**H17**		1														1
**H18**											1					1
**H19**														1		1
**H20**														1		1
**H21**										1						1
**H22**				1												1
**H23**								1								1
**H24**											1					1
**H25**										2		1				3
**H26**													1			1
**H27**						1										1
**H28**									1							1
**H29**					1											1
**H30**													1			1
**H31**		1														1
**H32**						1								1		2
**H33**												1				1
**H34**		1														1
**H35**		1	6	2	5	4	2	2								22
**H36**									1							1
**H37**									1							1
**H38**			1			1										2
**H39**				3												3
**H40**												1				1
**H41**				1												1
**H42**										1						1
**H43**	2	11	6	11	11	9	3	3		1				1		58
**H44**										1						1
**H45**			1													1
**H46**		2	1													3
**H47**					1											1
**H48**					1											1
**H49**										1						1
**H50**							1									1
**H51**										1						1
**H52**						1										1
**H53**														1		1
**H54**												1				1
**H55**														1		1
**H56**													1			1
**H57**						1										1
**H58**								1	1							2
**H59**								1								1
**H60**					1											1
**H61**														1		1
**H62**									1							1
**H63**					1											1
**H64**										1						1
**H65**												1				1
**H66**												1				1
**H67**															1	1
**H68**												1				1
**H69**										1				1		2
**H70**															1	1
**H71**													1			1
**Total**	3	22	17	18	21	20	8	9	5	11	3	9	7	8	2	163

H: Haplotype, Numbers show number of isolates. Total: Total No. of isolates, Two predominant haplotypes, H35 and H43 are highlighted in bold.

**Figure 2:**
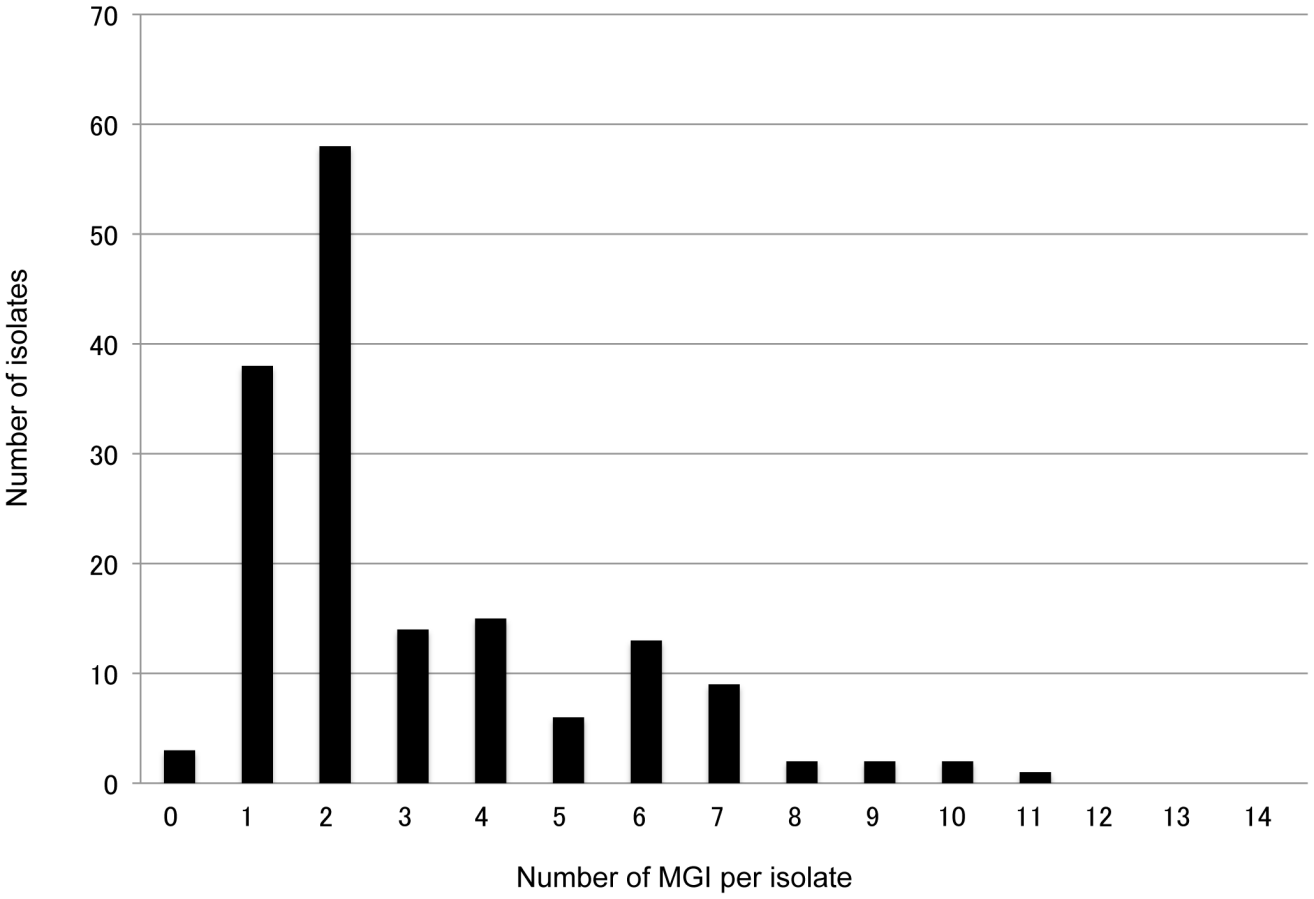
Frequency of MGI loci per isolate. A zero in the x-axis indicates that no MGI loci were observed in a particular isolate, that is, it represents a single clone infection isolate.

Instances where different sizes of alleles were observed in one locus, were regarded as multiple genotype infections (MGIs) - which were observed in some of the 14 microsatellite loci in 160 of the 163 isolates (98.2%). The frequencies of MGIs varied among the 14 loci (0.02 to 0.74; average: 0.22) (Table 2). We also examined the number of MGI loci per isolate. In the 163 isolates with 14 loci, the highest frequency of MGI loci per isolate was 2 (58 isolates). The frequencies decreased gradually according to the increase in the number of MGI loci (Fig. 2). The highest observed number of MGI loci per isolate was 11 (one isolate). The major alleles in each locus were used for population genetic analysis.

**Table 2:** Multiple Genotype Infection rate per locus of the South Korean *P. vivax* population.

Locus	No. of MGI isolates	MGI rate (%)
**MS1**	28	17.2
**MS2**	20	12.3
**MS3**	15	9.2
**MS4**	37	22.7
**MS5**	49	30.1
**MS6**	26	16.0
**MS7**	121	74.2
**MS8**	59	36.2
**MS9**	47	28.8
**MS10**	3	1.8
**MS12**	49	30.1
**MS15**	7	4.3
**MS16**	3	1.8
**MS20**	27	16.6
**Average**	35.1	21.5

MGI: Multiple genotype infection. Sample size: 163 isolates.

### Level of multilocus linkage disequilibrium of the South Korean *P. vivax* population

First, we estimated the level of multilocus LD using allelic data from all of the South Korean isolates (Table 3). When all haplotypes and unique haplotypes were both used in the analysis, significant multilocus LD (*P*<0.001) was observed in the *P. vivax* population of South Korea. Second, we calculated temporal changes to the levels of multilocus LD for each year, or 2–3 successive years when the number of isolates per year was less than 10 (Table 4). Significant multilocus LD (*P*<0.001) was again observed for all groups of isolates (Table 4). However, when all haplotypes were used in the analysis, the levels of multilocus LD gradually decreased over the study period: e.g., *I*_A_^S^ = 0.446 in 1994–1995 (25 isolates), *I*_A_^S^ = 0.390 in 2000–2001 (17 isolates), *I*_A_^S^ = 0.109 in 2006–2008 (17 isolates). These tendencies were also observed when unique haplotypes were used (Table 4). Significant multilocus LD (*P*<0.001) was observed when all haplotypes and unique haplotypes were both used. Thus it can be postulated that there has not been a recent epidemic expansion of any particular clones in the *P. vivax* population in South Korea [7], [8].

**Table 3:** Multilocus linkage disequilibrium in the South Korean *P. vivax* population from 1994 and 2008.

All Haplotypes		Unique Haplotypes Only	
No.	*I_A_^S^*	No.	*I_A_^S^*
163	0.356	71	0.143

Unique haplotypes show haplotypes excluding duplicates of any multiply represented infection. No. indicates the number of isolates for each measure. All values are significantly different from 0 (*P*<0.001).

**Table 4:** Multilocus linkage disequilibrium in the South Korean *P. vivax* populations.

Population	All Haplotypes		Unique Haplotypes Only	
	No.	*I_A_^S^*	No.	*I_A_^S^*
**1994–1995**	25	0.446	12	0.276
**1996**	17	0.524	7	0.416
**1997**	18	0.433	5	0.264
**1998**	21	0.466	7	0.238
**1999**	20	0.420	9	0.196
**2000–2001**	17	0.390	8	0.232
**2002–2003**	16	0.210	15	0.199
**2004–2005**	12	0.133	12	0.133
**2006–2008**	17	0.109	17	0.109

Unique haplotypes show haplotypes excluding duplicates of any multiply represented infection. No. indicates the number of isolates for each measure. All values are significantly different from 0 (*P*<0.001).

### Genetic differentiation of the South Korean *P. vivax* populations between successive years

The levels of genetic differentiation of the *P. vivax* population in South Korea for all pairs of years were evaluated by *F*_ST_ values (Table 5). Significant genetic differentiation was mainly observed between the groups of populations during the 1990s and the 2000s. It is noteworthy that the population of 2001 was genetically differentiated from the populations of 2003 and later. When the levels of genetic differentiation of the populations were evaluated between 2 successive years, significant genetic differentiation was observed between the populations of 2006 (7 isolates) and 2007 (8 isolates), although the sample size of each year was relatively small (<10 isolates) (*F*_ST_ = 0.124, *P*<0.05) (Table 5). Next, because the number of isolates in some years was relatively small (<10 isolates/year), we combined allelic data of the isolates of 2 or 3 successive years in order to increase the sample size of each group (>10 isolates/group), and reevaluated the levels of genetic differentiation. After the allelic data were combined, significant genetic differentiation was observed between the populations of 2000–2001 (17 isolates) and 2002–2003 (16 isolates) (*F*_ST_ = 0.052, *P*<0.01) (Table 6). On the contrary, no significant genetic differentiation was observed between the populations of 2005–2006 (16 isolates) and 2007–2008 (10 isolates) (*F*_ST_ = 0.017, *P*>0.01).

**Table 5:** Genetic differentiation (*F*_ST_) of pairwise comparisons between two years in the South Korean *P. vivax* populations.

Year	1994	1995	1996	1997	1998	1999	2000	2001	2002	2003	2004	2005	2006	2007
**1995**	−0.194													
**1996**	−0.185	0.007												
**1997**	−0.077	0.011	0.114											
**1998**	−0.196	−0.014	−0.023	0.035										
**1999**	−0.175	0.016	−0.045	0.122	−0.020									
**2000**	−0.269	−0.057	−0.074	0.049	−0.063	−0.065								
**2001**	−0.174	0.048	−0.065	0.206*	0.002**	−0.057	−0.068							
**2002**	−0.016	0.181**	0.047	0.375**	0.155*	0.051*	0.073	−0.005						
**2003**	−0.077	0.297**	0.073**	0.168*	0.081**	0.072*	0.027	0.077*	0.040					
**2004**	0.206	0.397*	0.326**	0.581**	0.419**	0.324**	0.302*	0.291**	0.118	0.112				
**2005**	−0.002	0.170**	0.125*	0.299**	0.176**	0.127**	0.088*	0.091*	0.000	−0.016	0.088			
**2006**	0.161	0.338**	0.266**	0.481**	0.345**	0.267**	0.245**	0.217**	0.073	0.113*	−0.055	0.035		
**2007**	0.002	0.161**	0.099*	0.304**	0.144*	0.089*	0.077	0.070	−0.040	−0.027	0.212*	−0.059	0.124*	
**2008**	0.250	0.406**	0.327*	0.627*	0.412*	0.319*	0.312	0.282**	0.169	0.142	0.211	0.007	0.093	0.090

* : 0.01<*P*<0.05

** : *P*<0.01

**Table 6:** Levels of genetic differentiation (*F*_ST_) between the South Korean *P. vivax* populations of successive groups of years.

Year	*F*_ST_	*P* value
**1994–1995 & 1996**	0.006	NS
**1999 & 2000–2001**	−0.044	NS
**2000–2001 & 2002–2003**	0.052	*P*<0.01
**2002–2003 & 2004–2005**	−0.007	NS
**2004–2005 & 2006–2008**	−0.031	NS
**2005–2006 & 2007–2008**	0.017	NS

NS: not significant.

### Population structure analysis of the South Korean *P. vivax* population by Bayesian approach

**Figure 3:**
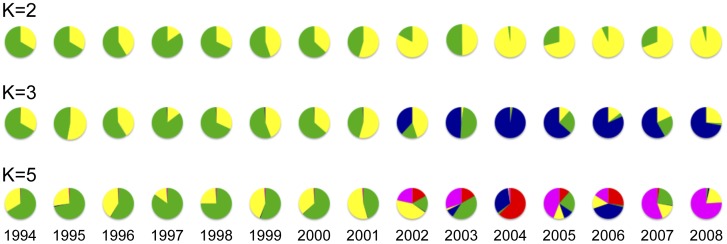
Proportion of ancestral populations among the South Korean *P. vivax* populations from 1994 to 2008. The colors represent the different ancestral populations of the South Korean *P. vivax* population (163 isolates) for *K* = 2, 3 and 5, estimated by STRUCTURE version 2.3.4 software [43]. These circular graphs were made based on results of the STRUCTURE analysis shown in Figure 1. The colors of the graphs correspond to those of Figure 1.

**Figure 4:**
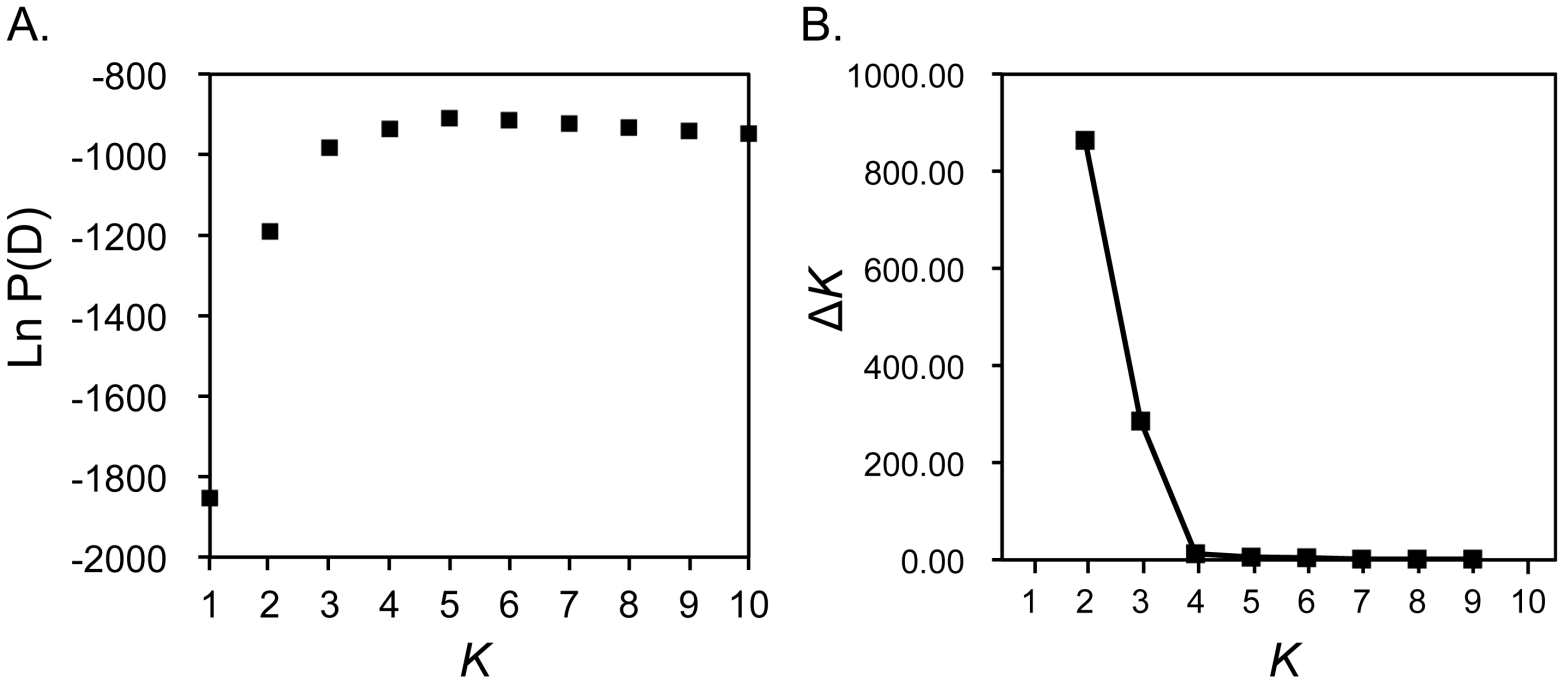
Estimation of population numbers (*K*) in the South Korean *P. vivax* population from 1994 to 2008. The population structure of the South Korean *P. vivax* population (163 isolates) from 1994 to 2008 was estimated by Bayesian approach using STRUCTURE software version 2.3.4, under the assumption of uncorrelated allele frequencies model [43]. (A) Averages of the log probability of the data [Ln P(D)] for each *K* value over 10 runs were plotted. (B) Δ*K* values for each *K* value were plotted. Δ*K* is the rate of change in Ln P(D) between successive *K* values [44].

The results of STRUCTURE analysis using an independent allele frequency model are shown in Figures 1 and 3. The highest value of Ln P(D) was observed at *K* = 5 (Fig. 4 A). This result suggested that the most appropriate population number (*K*) of *P. vivax* in South Korea is 5. However, the differences between Ln P(D) values of each *K* (3–10) were very small. In order to make the break in slope of the distribution of Ln P(D) salient at true *K*, we calculated value of Δ*K* using the method described by Evanno et al. [44]. The highest value of Δ*K* was observed at *K* = 2, and the second highest was observed at *K* = 3 (Fig. 4 B). These three *K* values (*K* = 2, 3 and 5) were thus adopted (Fig. 1 and 3). For *K* = 5, two genotypes (light green, yellow) were predominant from 1994 to 2002. Five genotypes (light green, blue, purple, red, yellow) coexisted from 2003 to 2008 (Fig. 1 and 3). For *K* = 2, two genotypes (light green, yellow) coexisted from 1994 to 2008. The light green genotype was predominant from 1994 to 2000, whereas the yellow genotype was predominant from 2001 to 2008 (Fig. 1 and 3). For *K* = 3, two genotypes (light green, yellow) coexisted from 1994 to 2001, while three genotypes (blue, light green, yellow) coexisted from 2002 to 2008 (Fig. 1 and 3). For all *K* values greater than 2, there was a drastic genetic change in the population structure of *P. vivax* in South Korea during 2002 and 2003. The results of STRUCTURE analysis, which used a model of correlated allele frequency among population, were almost the same as those of the analysis using the independent allele frequency model.

## Discussion

This is the second report on our 15-year-long longitudinal study on *P. vivax* population genetics in South Korea using highly polymorphic neutral markers. In the present study, we assessed characteristics of the population structure and temporal dynamics of parasite transmission in a more detailed and precise manner than was employed in our previous study, using the allelic data of 14 microsatellite DNA loci of the 163 isolates in South Korea (1994–2008).

The results of FSTAT analysis were basically consistent with the results of the STRUCTURE analysis based on the Bayesian approach (Fig. 1 and 3) [43], [44].

It is noteworthy that some negative *F*_ST_ values were observed in this study. According to Árnason and Pálsson, a negative *F*_ST_ value indicates great differences between two random individuals from the same population, rather than between two random individuals from different populations [45].

We estimated the population number, population structure and temporal dynamics of *P. vivax* transmission in South Korea using the STRUCTURE version 2.3.4 software [43], [44]. However, the estimation of the population number (*K*) using this method was problematic. When the log probability of the data [Ln P(D)] was used for estimating the most appropriate population number (*K*), 5 (*K* = 5) showed the highest value of Ln P(D) (Fig. 4 A). However, when Δ*K* was used, which is the rate of change in log probability, 2 (*K* = 2) showed the highest value of Δ*K* (Fig. 4 B). As a result, it was not possible to estimate true *K* in this analysis.

In our previous study using eBURST analysis [46], we found that two predominant haplotypes were observed from 1994 to 2005 and that new haplotypes had also been appearing since 1997 [33]. In a basic sense, our present study supports these previous findings. When the population number was assumed to be 2 (*K* = 2), two different genotypes (light green, yellow) coexisted over the study period, but the frequencies of the two genotypes changed around 2001: the light green genotype was predominant until 2000, while yellow has been predominant since 2001 (Fig. 1 and 3). In contrast, when the population number was assumed to be greater than or equal to 3 (*K*≥3), two different genotypes coexisted until 2001, and thereafter, new genotypes appeared and were predominant from 2002. In the present study, when *K* was ≥3, the two genotypes that were predominantly observed until 2001 corresponded to groups 1 and 2 of the *P. vivax* populations in South Korea of the previous study [33].

Honma et al. also conducted STRUCTURE analysis of the *P. vivax* population in South Korea (1997–2000, 2007) using allelic data of 13 microsatellite DNA markers [35]. The Honma et al. study used 9 out of the 13 microsatellite DNA markers that were used in the present study (MS3, 5, 6, 8, 9, 10, 15, 16, 20).. They observed the highest value of Ln P(D) when *K* was 5 (*K* = 5) and also the highest value of Δ*K* when *K* was 3 (*K* = 3). For these reasons, these two *K* values were adopted. They found a drastic change in the population structure of *P. vivax* between the groups of 1997–2000 and 2007 at both *K* values. The latter group showed an increase in genetic diversity. However, the year in which the parasite population changed could not be estimated because of their sampling limitation. In the present study, we estimated that the parasite population structure in South Korea underwent a drastic change during 2002 and 2003. This change of the population structure was observed at *K* = 3–10.

Some of the previous studies reported that the level of genetic diversity in the South Korean *P. vivax* population has been increasing in recent years [33], [34]. However, the multilocus LD levels of the population were shown to have been gradually decreasing: *I*_A_^S^ = 0.584, *P*<0.001 for 1994–1998; *I*_A_^S^ = 0.315, *P*<0.001 for 1999–2003; *I*_A_^S^ = 0.140, *P*<0.001 for 2004–2008 [30]. Honma et al. showed a similar tendency in South Korean population groups of 1997–2000 and 2007 [35]. In the present study, we performed multilocus LD analysis for each year or 2–3 successive years and demonstrated that the levels of multilocus LD decreased successively over the study period with few exceptions. Moreover, the level of multilocus LD drastically decreased from the group of 2001–2002 to that of 2003–2004 (Table 4). These results were consistent with the results of the STRUCTURE analysis in that the variation of the alleles in the population suddenly increased from 2002. These changes might enhance outbreeding between different genotypes in the population.

In the case of *P. falciparum* populations, Tanabe et al. observed a strong negative correlation between “within-population genetic diversity” and “geographic distance from sub-Saharan Africa over Africa, Asia, and Oceania” [47]. In contrast, other studies suggested that the levels of genetic diversity of the parasite populations are higher in high-transmission areas and lower in low-transmission areas [7], [8]. Some exceptions, however, have been reported [48]. In contrast, a negative correlation as was observed with *P. falciparum*, has thus far never been reported with *P. vivax*. Likewise, there have been no reports of a correlation between the levels of genetic diversity and the levels of malaria endemicity in the *P. vivax* population. Relatively high levels of genetic diversity are reported even in low transmission areas, such as Sri Lanka [37]. This feature could be attributed to relapse, owing to hypnozoites in the liver of the vivax malaria patient.

In South Korea, the number of vivax malaria cases fluctuated between 864 and 2,227 cases per year from 2004 to 2011. This suggested dramatic changes in endemicity in the endemic area [1]. Moreover, previous studies suggested that the recombination rate of the *P. vivax* population in South Korea was very low [33]. Therefore, the increase in genetic diversity and the decrease of LD levels of the *P. vivax* population in South Korea cannot be attributed to increased outbreeding between different genotypes. Thus, we would prefer to attribute it to the introduction of a genetically different *P. vivax* population (or populations) from other sources.

The majority of malaria patients were South Korean soldiers or veterans who served in or near the DMZ, and some civilians who lived in the same area. The width of the DMZ is 4 kilometers or less [49], [50]. Given that the *Anopheles sinensis*, the main vector species of vivax malaria in South Korea has been demonstrated to fly up to 12 km in one night [49], the geographical origin of the other population sources is probably North Korea. Furthermore, the reported number of vivax malaria cases in North Korea was many times higher than that in South Korea [1]. Why did the parasite population structure in South Korea change drastically during 2002 and 2003 and what happened to the parasite population in that time? One possible scenario is that the genetic diversity of the parasite population in North Korea increased and the consequent outbreeding between different genotypes could have also occurred frequently in the endemic area in 2001, because the number of *P. vivax* cases in the North Korea was the highest (300,000 cases) in that year [1]. Then, this genetically diverse population could have been introduced into South Korea by *Anopheles* mosquitoes flying over the DMZ in 2001. Thereafter, those new genotypes started to appear in South Korean patients from 2002 due to the 8–13-month incubation period of *P. vivax* on the Korean peninsula [26]. That the effect of high transmission in North Korea was not reflected in higher transmission in South Korea in the following year is probably due to the use of chloroquine and primaquine for chemoprophylaxis by South Korean soldiers [50]. Although the proportion of civilian patients in South Korea increased, in 2001 and 2002, about 60% of the patients were soldiers or veterans [51]. Therefore, the number of vivax cases in South Korea did not increase during 2002 and 2003 but the genetic profile of the population changed drastically, owing to the results of genetic drift such as the founder effect (introduction by infected *Anopheles* mosquitoes) and/or genetic bottleneck (by chemoprophylaxis).

Chemoprophylaxis was provided to South Korean soldiers before 2001, but not to all soldiers [50]. For example, chloroquine prophylaxis was emphasized for the soldiers who served in Paju, Yeoncheon, and Cheolwon counties, whereas it was not provided to the soldiers who served in Pocheon and Hwacheon counties in 1999. These differences might influence the population structure of *P. vivax* in South Korea. Moreover, relapse and asymptomatic parasite carriage might play an important role in the population change. However, such factors have been implicated in the parasite population since 1993, with the reemergence of vivax malaria in South Korea. Thus, relapse and asymptomatic parasite carriage can be thought to have had little influence on the population change. The possibility exists, although it is mere speculation, that both the drastic change in the number of North Korean vivax malaria cases and the use of chemoprophylaxis by South Korean soldiers have influenced the structure of the parasite population in South Korea.

In conclusion, molecular genetic epidemiology using polymorphic DNA markers of the *P. vivax* genome is a very useful tool for assessing the population structure and temporal dynamics of the transmission of the parasites, the knowledge of which may lead to the effective control of vivax malaria in endemic areas.

## Supporting Information

Figure S1 **Genetic diversity of the** ***P. vivax*** **population in South Korea for years 1994–2000 and 2001–2008.** A: Average number of alleles ± SE, *H*_E_: Average expected heterozygosity ± SE. Numbers (without parentheses) represent the number of haplotypes observed for each year. The numbers in parentheses represent the number of isolates. n: represents the total number of isolates. *H*_E_ values for each locus were calculated using *H*_E_ = [n/(n−1)] [1−∑*P*_i_^2^], where *P*_i_ is the frequency of the i^th^ allele. The graph was made based on numbers of reported vivax malaria cases in South Korea. The data were obtained from the World Malaria Report 2012 (WHO) [1]. This figure corresponds to Figure 1 in our previous study [33].
(TIF)

Figure S2 **Genetic diversity of the** ***P. vivax*** **population in South Korea for years 1994–1998, 1999–2003 and 2004–2008.** A: Average number of alleles ± SE, *H*_E_: Average expected heterozygosity ± SE. Numbers (without parentheses) represent the number of haplotypes observed for each year. The numbers in parentheses represent the number of isolates. n: represents the total number of isolates. *H*_E_ values for each locus were calculated using *H*_E_ = [n/(n−1)] [1−∑*P*_i_^2^], where *P*_i_ is the frequency of the i^th^ allele. The graph was made based on numbers of reported vivax malaria cases in South Korea. The data were obtained from the World Malaria Report 2012 (WHO) [1]. This figure corresponds to Figure 3 in our previous study [33].
(TIF)

Table S1 **List of 14 microsatellite DNA loci and genetic diversity of the South Korean** ***P. vivax*** **population.** Chr: Chromosome No, A: Number of alleles, *H*_E_: Expected heterozygosity, n: Number of isolates, *H*_E_ values for each locus were calculated using *H*_E_ = [n/(n−1)] [1−∑*P*_i_^2^], where *P*_i_ is the frequency of the i^th^ allele. § Two genes are encoding a hypothetical protein and a merozite surface protein 7.
(XLS)

Table S2 **Allelic composition of each haplotype of the South Korean** ***P. vivax*** **population.** Each number represents type of alleles in each locus.
(XLS)

## References

[1] World Health Organization (2012) World Malaria Report 2012. Geneva: WHO.

[2] Guerra CA, Howes RE, Patil AP, Gething PW, Van Boeckel TP, et al (2010) The international limits and population at risk of *Plasmodium vivax* transmission in 2009. PLoS Negl Trop Dis 4: e774. 20689816 10.1371/journal.pntd.0000774 PMC2914753

[3] World Health Organization (2010) Global report on antimalarial drug efficacy and drug resistance: 2000–2010. Geneva: WHO.

[4] Tan LK, Yacoub S, Scott S, Bhagani S, Jacobs M (2008) Acute lung injury and other serious complications of *Plasmodium vivax* malaria. Lancet Infect Dis 8: 449–454. 18582837 10.1016/S1473-3099(08)70153-1

[5] Mueller I, Galinski MR, Baird JK, Carlton JM, Kochar DK, et al (2009) Key gaps in the knowledge of *Plasmodium vivax*, a neglected human malaria parasite. Lancet Infect Dis 9: 555–566. 19695492 10.1016/S1473-3099(09)70177-X

[6] Enserink M (2010) As challenges change, so does science. Science 328: 843. 20466916 10.1126/science.328.5980.843

[7] Iwagami M, Rivera PT, Villacorte EA, Escueta AD, Hatabu T, et al (2009) Genetic diversity and population structure of *Plasmodium falciparum* in the Philippines. Malar J 8: 96. 19422722 10.1186/1475-2875-8-96 PMC2685811

[8] Anderson TJ, Haubold B, Williams JT, Estrada-Franco JG, Richardson L, et al (2000) Microsatellite markers reveal a spectrum of population structures in the malaria parasite *Plasmodium falciparum* . Mol Biol Evol 17: 1467–1482. 11018154 10.1093/oxfordjournals.molbev.a026247

[9] Machado RL, Povoa MM, Calvosa VS, Ferreira MU, Rossit AR, et al (2004) Genetic structure of *Plasmodium falciparum* populations in the Brazilian Amazon region. J Infect Dis 190: 1547–1555. 15478058 10.1086/424601

[10] Anthony TG, Conway DJ, Cox-Singh J, Matusop A, Ratnam S, et al (2005) Fragmented population structure of *Plasmodium falciparum* in a region of declining endemicity. J Infect Dis 191: 1558–1564. 15809916 10.1086/429338

[11] Mu J, Joy DA, Duan J, Huang Y, Carlton J, et al (2005) Host switch leads to emergence of *Plasmodium vivax* malaria in humans. Mol Biol Evol 22: 1686–1693. 15858201 10.1093/molbev/msi160

[12] Jongwutiwes S, Putaporntip C, Iwasaki T, Ferreira MU, Kanbara H, et al (2005) Mitochondrial genome sequences support ancient population expansion in *Plasmodium vivax* . Mol Biol Evol 22: 1733–1739. 15901839 10.1093/molbev/msi168 PMC1224720

[13] Cornejo OE, Escalante AA (2006) The origin and age of *Plasmodium vivax* . Trends Parasitol 22: 558–563. 17035086 10.1016/j.pt.2006.09.007 PMC1855252

[14] Imwong M, Nair S, Pukrittayakamee S, Sudimack D, Williams JT, et al (2007) Contrasting genetic structure in *Plasmodium vivax* populations from Asia and South America. Int J Parasitol 37: 1013–1022. 17442318 10.1016/j.ijpara.2007.02.010

[15] Ferreira MU, Karunaweera ND, da Silva-Nunes M, da Silva NS, Wirth DF, et al (2007) Population structure and transmission dynamics of *Plasmodium vivax* in rural Amazonia. J Infect Dis 195: 1218–1226. 17357061 10.1086/512685

[16] Gunawardena S, Karunaweera ND, Ferreira MU, Phone-Kyaw M, Pollack RJ, et al (2010) Geographic structure of *Plasmodium vivax*: microsatellite analysis of parasite populations from Sri Lanka, Myanmar, and Ethiopia. Am J Trop Med Hyg 82: 235–242. 20133999 10.4269/ajtmh.2010.09-0588 PMC2813164

[17] Van den Eede P, Van der Auwera G, Delgado C, Huyse T, Soto-Calle VE, et al (2010) Multilocus genotyping reveals high heterogeneity and strong local population structure of the *Plasmodium vivax* population in the Peruvian Amazon. Malar J 9: 151. 20525233 10.1186/1475-2875-9-151 PMC2898784

[18] Chenet SM, Schneider KA, Villegas L, Escalante AA (2012) Local population structure of *Plasmodium*: impact on malaria control and elimination. Malar J 11: 412. 23232077 10.1186/1475-2875-11-412 PMC3538601

[19] Koepfli C, Timinao L, Antao T, Barry AE, Siba P, et al (2013) A Large *Plasmodium vivax* Reservoir and Little Population Structure in the South Pacific. PLoS One 8: e66041. 23823758 10.1371/journal.pone.0066041 PMC3688846

[20] Kho WG, Park YH, Chung JY, Kim JP, Hong ST, et al (1999) Two new genotypes of *Plasmodium vivax* circumsporozoite protein found in the Republic of Korea. Korean J Parasitol 37: 265–270. 10634043 10.3347/kjp.1999.37.4.265 PMC2733204

[21] Kho WG, Chung JY, Sim EJ, Kim DW, Chung WC (2001) Analysis of polymorphic regions of *Plasmodium vivax* Duffy binding protein of Korean isolates. Korean J Parasitol 39: 143–150. 11441501 10.3347/kjp.2001.39.2.143 PMC2721091

[22] Chung JY, Chun EH, Chun JH, Kho WG (2003) Analysis of the *Plasmodium vivax* apical membrane antigen-1 gene from re-emerging Korean isolates. Parasitol Res 90: 325–329. 12690444 10.1007/s00436-002-0777-2

[23] Kim SH, Hwang SY, Shin JH, Moon CS, Kim DW, et al (2009) Molecular genetic characterization of the merozoite surface protein 1 Gene of *Plasmodium vivax* from reemerging Korean isolates. Clin Vaccine Immunol 16: 733–738. 19261779 10.1128/CVI.00493-08 PMC2681600

[24] Hwang SY, Kim SH, Kho WG (2009) Genetic characteristics of polymorphic antigenic markers among Korean isolates of *Plasmodium vivax* . Korean J Parasitol 47 Suppl: S51–S58. 19885335 10.3347/kjp.2009.47.S.S51 PMC2769223

[25] Iwagami M, Hwang SY, Fukumoto M, Hayakawa T, Tanabe K, et al (2010) Geographical origin of *Plasmodium vivax* in the Republic of Korea: haplotype network analysis based on the parasite's mitochondrial genome. Malar J 9: 184. 20576165 10.1186/1475-2875-9-184 PMC2908639

[26] Chai JY (1999) Re-emerging *Plasmodium vivax* malaria in the Republic of Korea. Korean J Parasitol 37: 129–143. 10507220 10.3347/kjp.1999.37.3.129 PMC2733142

[27] Ree HI (2000) Unstable vivax malaria in Korea. Korean J Parasitol 38: 119–138. 11002647 10.3347/kjp.2000.38.3.119 PMC2721191

[28] Shin EH, Guk SM, Kim HJ, Lee SH, Chai JY (2008) Trends in parasitic diseases in the Republic of Korea. Trends Parasitol 24: 143–150. 18255338 10.1016/j.pt.2007.12.003

[29] Chai IH, Lim GI, Yoon SN, Oh WI, Kim SJ, et al (1994) [Occurrence of tertian malaria in a male patient who has never been abroad]. Korean J Parasitol 32: 195–200 [Article in Korean, English abstract available]. 7953245 10.3347/kjp.1994.32.3.195

[30] Park JW, Jun G, Yeom JS (2009) *Plasmodium vivax* malaria: status in the Republic of Korea following reemergence. Korean J Parasitol 47 Suppl: S39–S50. 19885334 10.3347/kjp.2009.47.S.S39 PMC2769212

[31] Han ET, Lee DH, Park KD, Seok WS, Kim YS, et al (2006) Reemerging vivax malaria: changing patterns of annual incidence and control programs in the Republic of Korea. Korean J Parasitol 44: 285–294. 17170570 10.3347/kjp.2006.44.4.285 PMC2559126

[32] Feighner BH, Pak SI, Novakoski WL, Kelsey LL, Strickman D (1998) Reemergence of *Plasmodium vivax* malaria in the Republic of Korea. Emerg Infect Dis 4: 295–297. 9621202 10.3201/eid0402.980219 PMC2640128

[33] Iwagami M, Fukumoto M, Hwang SY, Kim SH, Kho WG, et al (2012) Population structure and transmission dynamics of *Plasmodium vivax* in the Republic of Korea based on microsatellite DNA analysis. PLoS Negl Trop Dis 6: e1592. 22509416 10.1371/journal.pntd.0001592 PMC3317904

[34] Choi YK, Choi KM, Park MH, Lee EG, Kim YJ, et al (2010) Rapid dissemination of newly introduced *Plasmodium vivax* genotypes in South Korea. Am J Trop Med Hyg 82: 426–432. 20207868 10.4269/ajtmh.2010.09-0245 PMC2829904

[35] Honma H, Kim JY, Palacpac NM, Mita T, Lee W, et al (2011) Recent increase of genetic diversity in *Plasmodium vivax* population in the Republic of Korea. Malar J 10: 257. 21899730 10.1186/1475-2875-10-257 PMC3176257

[36] Sambrook J, Russell DW (2001) Molecular Cloning: A Laboratory Manual. 3rd edition. New York: Cold Spring Harbor Laboratory Press. pp. 6.4–6.12.

[37] Karunaweera ND, Ferreira MU, Hartl DL, Wirth DF (2007) Fourteen polymorphic microsatellite DNA markers for the human malaria parasite *Plasmodium vivax* . Mol Ecol Notes 7: 172–175.

[38] Smith JM, Smith NH, O'Rourke M, Spratt BG (1993) How clonal are bacteria? Proc Natl Acad Sci USA 90: 4384–4388. 8506277 10.1073/pnas.90.10.4384 PMC46515

[39] Hudson RR (1994) Analytical results concerning linkage disequilibrium in models with genetic transformation and recombination. J Evol Biol 7: 535–548.

[40] Haubold B, Hudson RR (2000) LIAN 3.0: detecting linkage disequilibrium in multilocus data. Linkage Analysis. Bioinformatics 16: 847–848. 11108709 10.1093/bioinformatics/16.9.847

[41] Weir BS, Cockerham CC (1984) Estimating F-statistics for the analysis of population structure. Evolution 38: 1358–1370. 28563791 10.1111/j.1558-5646.1984.tb05657.x

[42] Goudet J (1995) FSTAT (version 1.2): a computer program to calculate F statistics. J Hered 86: 485–486.

[43] Pritchard JK, Stephens M, Donnelly P (2000) Inference of population structure using multilocus genotype data. Genetics 155: 945–959. 10835412 10.1093/genetics/155.2.945 PMC1461096

[44] Evanno G, Regnaut S, Goudet J (2005) Detecting the number of clusters of individuals using the software STRUCTURE: a simulation study. Mol Ecol 14: 2611–2620. 15969739 10.1111/j.1365-294X.2005.02553.x

[45] Árnason E, Pálsson S (1996) Mitochondrial cytochrome *b* DNA sequence variation of Atlantic cod *Gadus morhua* from Norway. Mol Ecol 5: 715–724. 10.1111/j.1601-5223.1998.00037.x 9868927

[46] Feil EJ, Li BC, Aanensen DM, Hanage WP, Spratt BG (2004) eBURST: inferring patterns of evolutionary descent among clusters of related bacterial genotypes from multilocus sequence typing data. J Bacteriol 186: 1518–1530. 14973027 10.1128/JB.186.5.1518-1530.2004 PMC344416

[47] Tanabe K, Mita T, Jombart T, Eriksson A, Horibe S, et al (2010) *Plasmodium falciparum* accompanied the human expansion out of Africa. Curr Biol 20: 1283–1289. 20656209 10.1016/j.cub.2010.05.053

[48] Branch OH, Sutton PL, Castro JC, Barnes C, Hussin J, et al (2011) *Plasmodium falciparum* genetic diversity maintained and amplified over 5 years of a low transmission endemic in the Peruvian Amazon. Mol Biol Evol 28: 1973–1986. 21109587 10.1093/molbev/msq311 PMC3112368

[49] Cho SH, Lee HW, Shin EH, Lee HI, Lee WG, et al (2002) A mark-release-recapture experiment with *Anopheles sinensis* in the northern part of Gyeonggi-do, Korea. Korean J Parasitol 40: 139–148. 12325443 10.3347/kjp.2002.40.3.139 PMC2721040

[50] Park JW, Klein TA, Lee HC, Pacha LA, Ryu SH, et al (2003) Vivax malaria: a continuing health threat to the Republic of Korea. Am J Trop Med Hyg 69: 159–167. 13677372

[51] Park JW, Jun G, Yeom JS (2009) *Plasmodium vivax* malaria: status in the Republic of Korea following reemergence. Korean J Parasitol 47 Suppl: S39–50. 19885334 10.3347/kjp.2009.47.S.S39 PMC2769212

