# Supplementary figures and images for: Microsatellite DNA Analysis Revealed a Drastic Genetic Change of Plasmodium vivax Population in the Republic of Korea During 2002 and 2003
Source: PLoS Negl Trop Dis. 2013 Oct 31;7(10):e2522. doi: 10.1371/journal.pntd.0002522 (PMC3814342; doi:10.1371/journal.pntd.0002522)

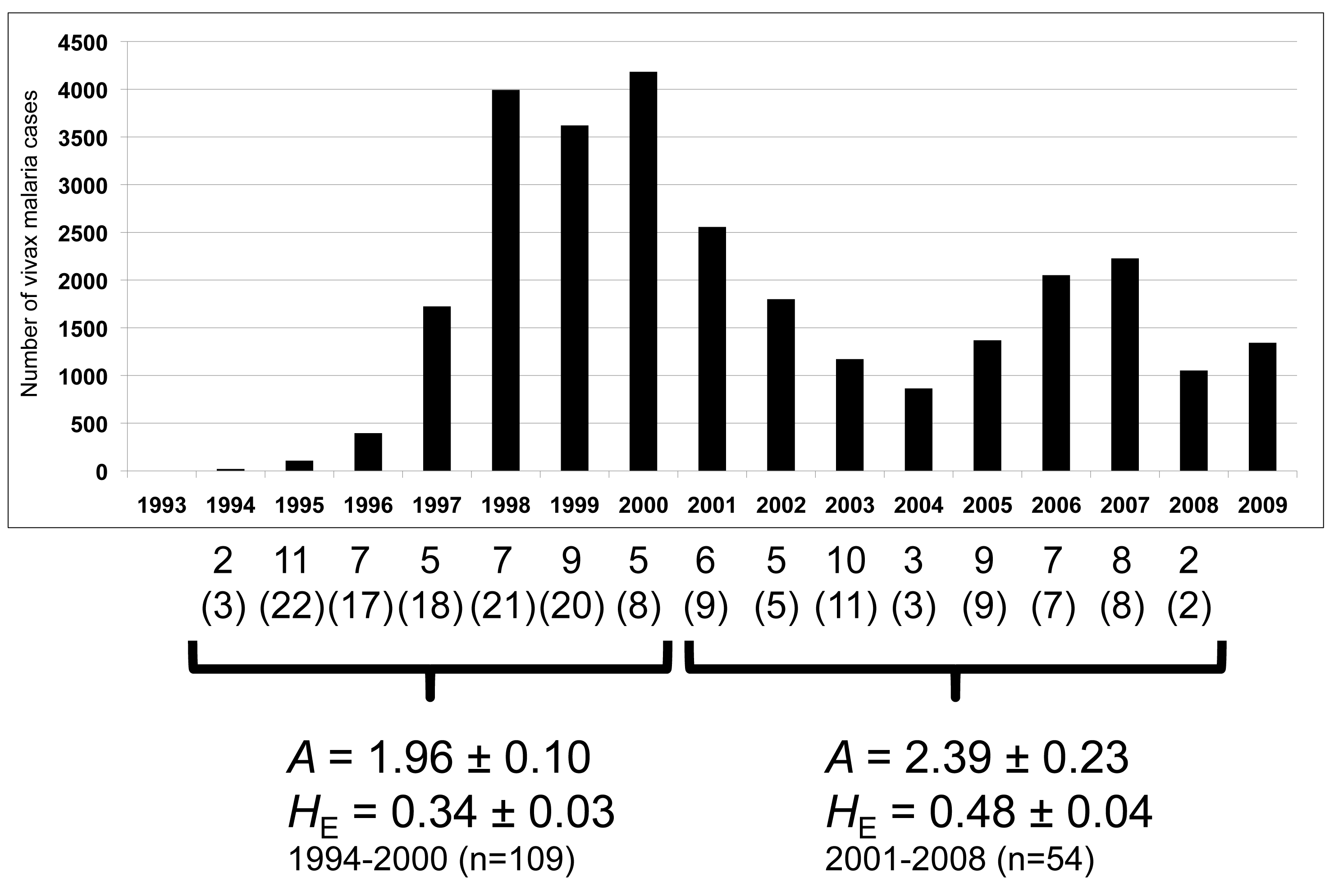

Supplement: Figure S1 — Genetic diversity of the P. vivax population in South Korea for years 1994–2000 and 2001–2008. A: Average number of alleles ± SE, H E: Average expected heterozygosity ± SE. Numbers (without parentheses) represent the number of haplotypes observed for each year. The numbers in parentheses represent the number of isolates. n: represents the total number of isolates. H E values for each locus were calculated using H E = [n/(n−1)] [1−∑P i 2], where P i is the frequency of the ith allele. The graph was made based on numbers of reported vivax malaria cases in South Korea. The data were obtained from the World Malaria Report 2012 (WHO) [1]. This figure corresponds to Figure 1 in our previous study [33]. (TIF) [file pntd.0002522.s001.tif]

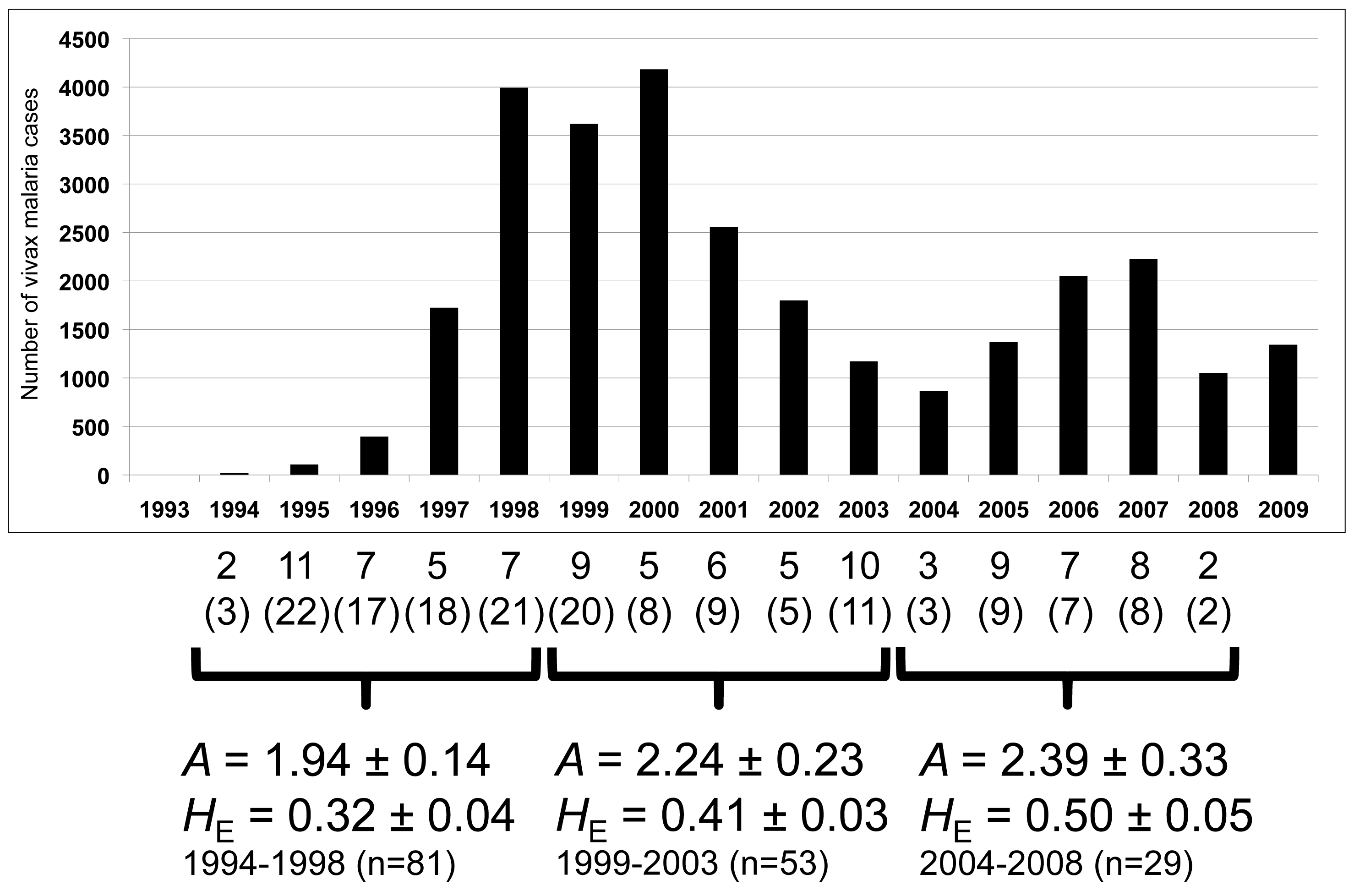

Supplement: Figure S2 — Genetic diversity of the P. vivax population in South Korea for years 1994–1998, 1999–2003 and 2004–2008. A: Average number of alleles ± SE, H E: Average expected heterozygosity ± SE. Numbers (without parentheses) represent the number of haplotypes observed for each year. The numbers in parentheses represent the number of isolates. n: represents the total number of isolates. H E values for each locus were calculated using H E = [n/(n−1)] [1−∑P i 2], where P i is the frequency of the ith allele. The graph was made based on numbers of reported vivax malaria cases in South Korea. The data were obtained from the World Malaria Report 2012 (WHO) [1]. This figure corresponds to Figure 3 in our previous study [33]. (TIF) [file pntd.0002522.s002.tif]
